# Increased Actin Polymerization and Stabilization Interferes with Neuronal Function and Survival in the AMPKγ Mutant *Loechrig*

**DOI:** 10.1371/journal.pone.0089847

**Published:** 2014-02-25

**Authors:** Mandy Cook, Bonnie J. Bolkan, Doris Kretzschmar

**Affiliations:** Oregon Institute of Occupational Health Sciences, Oregon Health & Sciences University, Portland, Oregon, United States of America; Institut de Génétique et Développement de Rennes, France

## Abstract

*loechrig (loe)* mutant flies are characterized by progressive neuronal degeneration, behavioral deficits, and early death. The mutation is due to a P-element insertion in the gene for the γ-subunit of the trimeric AMP-activated protein kinase (AMPK) complex, whereby the insertion affects only one of several alternative transcripts encoding a unique neuronal isoform. AMPK is a cellular energy sensor that regulates a plethora of signaling pathways, including cholesterol and isoprenoid synthesis via its downstream target hydroxy-methylglutaryl (HMG)-CoA reductase. We recently showed that *loe* interferes with isoprenoid synthesis and increases the prenylation and thereby activation of RhoA. During development, RhoA plays an important role in neuronal outgrowth by activating a signaling cascade that regulates actin dynamics. Here we show that the effect of *loe/*AMPKγ on RhoA prenylation leads to a hyperactivation of this signaling pathway, causing increased phosphorylation of the actin depolymerizating factor cofilin and accumulation of filamentous actin. Furthermore, our results show that the resulting cytoskeletal changes in *loe* interfere with neuronal growth and disrupt axonal integrity. Surprisingly, these phenotypes were enhanced by expressing the Slingshot (SSH) phosphatase, which during development promotes actin depolymerization by dephosphorylating cofilin. However, our studies suggest that in the adult SSH promotes actin polymerization, supporting *in vitro* studies using human SSH1 that suggested that SSH can also stabilize and bundle filamentous actin. Together with the observed increase in SSH levels in the *loe* mutant, our experiments suggest that in mature neurons SSH may function as a stabilization factor for filamentous actin instead of promoting actin depolymerization.

## Introduction

AMP-activated protein kinase (AMPK) is a protein complex consisting of a catalytic α-subunit and two regulatory subunits, β and γ. AMPK has first been described due to its role in regulating energy metabolism by promoting energy generating pathways, like fatty acid oxidation and glycolysis, and inhibiting energy demanding processes, like fatty acid and cholesterol synthesis, in the case of ATP depletion [1]. But besides these more general functions in metabolism, AMPK has also been shown to regulate aspects of protein synthesis, cell growth, and cell polarity [2], [3]. Therefore it is not surprising that AMPK is an evolutionary conserved protein that is expressed in all tissues, including the brain [4]. However, the combination of various isoforms for the three subunits, that are either produced from different genes or by alternative splicing [3], allows tissue and cellular specificity; for example the γ1 subunit is highly expressed in neurons but not in astrocytes in mice [5] whereas the γ3 subunit is mostly expressed in glycolytic skeletal muscle [6]. Although *Drosophila* also expresses several isoforms for each subunit, they are solely encoded by alternative transcripts and not by separate genes [7]. For the γ subunit, so far 16 alternative transcripts have been described in flies that encode six different protein isoforms (flybase.com; [8]). The P-element insertion in the *loe* mutant affects one of these transcripts which encodes a protein isoform that contains a unique N-terminus and that is required in the nervous system [9].

Although degeneration and vacuole formation can be detected in some parts of the larval brain of *loe* mutant flies, the adult brain appears to develop normal [10]. However 2–3 d after eclosion of the adult, spongiform lesions can be detected in these animals that increase in size and number with further aging [9]. Genetic interactions with *columbus (clb)*, the *Drosophila* orthologue of HMG-CoA reductase confirmed that, as in vertebrates, HMG-CoA reductase is negatively regulated by AMPK [9]. HMG-CoA reductase is known as a key factor in cholesterol synthesis, which does not occur *de novo* in flies [11], and isoprenoid synthesis, which is conserved in *Drosophila* [12]. Well-known targets of isoprenylation are small GTPases, like Ras, Rab, Rac, and Rho, which through the addition of these lipid moieties can associate with membranes and subsequently be activated [13]. The connection between *loe* and the isoprenoid pathway was confirmed by experiments showing that altering isoprenoid levels, either genetically or by pharmacological means, affected the severity of *loe* associated phenotypes and *loe* flies show increased levels of isoprenylated RhoA [14]. In addition, increased RhoA levels enhanced the degeneration observed in *loe*, whereas decreased amounts suppressed it. Together with the finding that induction of constitutively active RhoA can induce degeneration [14], these findings suggested that the hyperactivation of RhoA plays an important part in causing the progressive degeneration that occurs in the *loe* mutant.

Rho-GTPases are important regulators of actin dynamics, thereby affecting many functions of the cell. In neurons, Rho proteins have been shown to regulate axon formation and axonal guidance by coupling guidance clues with cytoskeletal rearrangements in the growth cone whereby different family members can have opposing functions [15]. Whereas Rac1 and Cdc42 generally induce neurite outgrowth, RhoA activation can result in growth cone collapse or promote forward progression, depending on its downstream effectors [15]–[17]. Rho-GTPases play such a major role in growth cone dynamics by affecting all aspects of the actin cycle, including the assembly and disassembly of filamentous (F-) actin through their effects on different downstream kinases [15]. Whereas the effects of RhoA are mostly mediated by the activation of Rho-associated kinases (ROCKs), Cdc42 and Rac signal through p21-activated kinases (PAKs) and several other kinases [17]. Both, ROCKs and PAKs, can then phosphorylate Lim kinase (LIMK) which in turn phosphorylates and thus inactivates cofilin, a key factor in actin polymerization [18]. Although *Drosophila* has less family members than vertebrates for some of these components, the same signaling cascade regulates axonal outgrowth in flies and the loss of cofilin or manipulations of its upstream regulators result in severe axon growth defects [19].

Though changes in the actin cytoskeleton have been connected with neuronal survival and neurodegenerative diseases much less is known about the involved pathways and mechanisms. Abnormal PAK activation was observed in the brains of patients with Alzheimer’s Disease (AD) and in cultured neurons treated with Aβ oligomers [20], [21], which are crucial in the pathology of AD. ROCK has been implicated in the processing of the Amyloid Precursor Protein (APP), from which the Aβ fragments are produced, and treatment with ROCK inhibitors reduced Aβ levels [22]. In addition, an increase in phosphorylated LIMK has been reported in brain areas affected by AD [23]. Experiments in cell culture and in *Drosophila* have suggested that tau and Aβ can activate LIMK and cofilin and promote the accumulation of F-actin [24], however the upstream activators have not been identified. Here we show that the *loe/*AMPKγ mutation causes an upregulation of the Rho/LIMK pathway, resulting in an increase in phosphorylated cofilin and the accumulation of F-actin. In addition, our experiments suggest that Slingshot, which promotes actin depolymerization during neuronal outgrowth, mostly acts as an actin stabilizing factor in the adult nervous system.

## Results

### The Effect of RhoA on Neuronal Survival in *Loe* is Mediated by ROCK and LIMK

**Figure 1:**
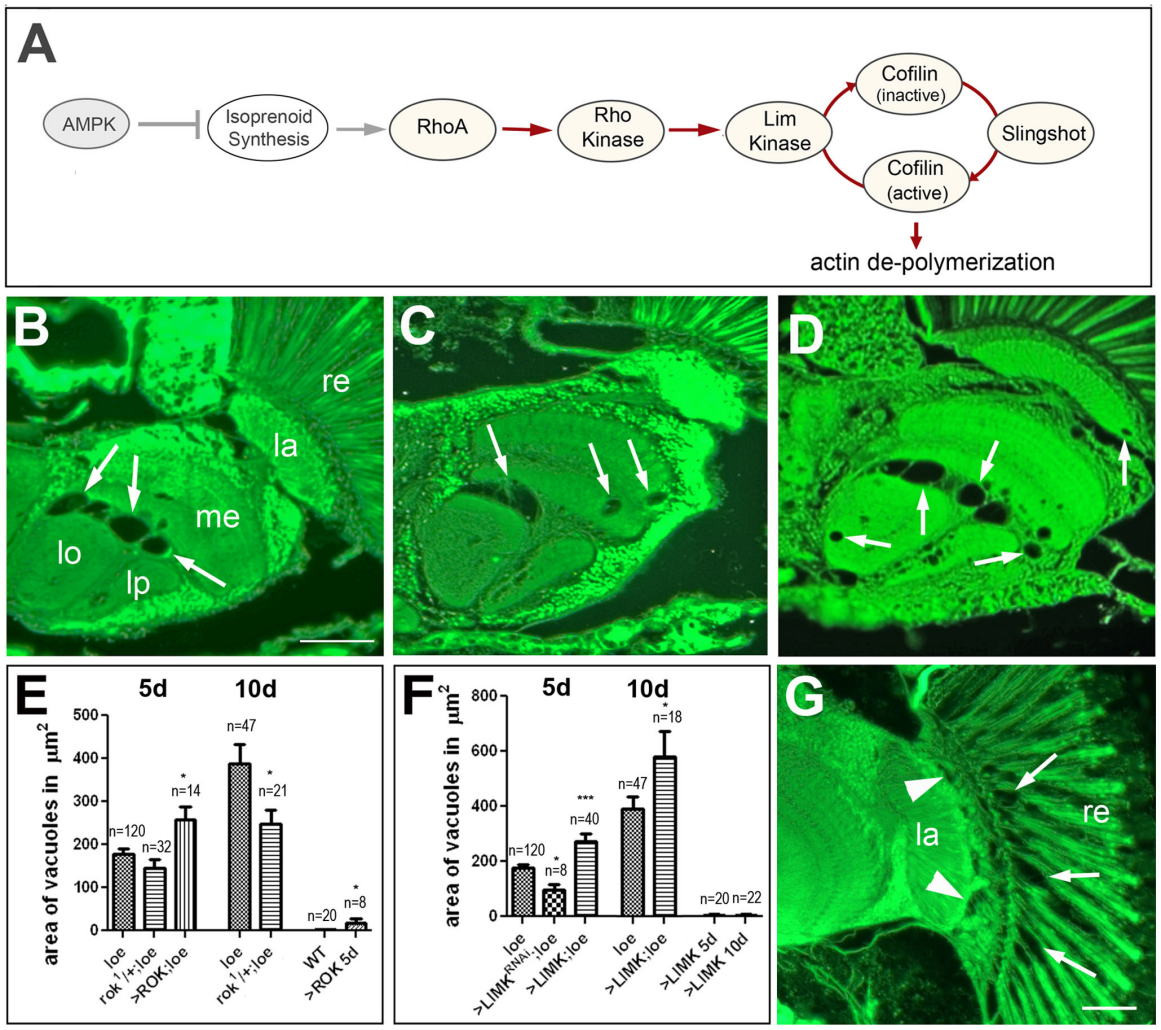
*loe* interferes with the RhoA/LIMK pathway. **A.** Schematic of the RhoA/LIMK pathway. AMPK negatively regulates isoprenoid synthesis. Isoprenylation allows RhoA’s association with membranes where it can subsequently be activated and interact with its downstream targets. **B.** Paraffin head section of a 10 d old homozygous *loe* fly reveals severe vacuolization (arrows). **C.** An age-matched female *rok^1^*/+;*loe/loe* fly shows less lesions. **D.** In contrast, a 10 d old female *loe* fly expressing LIMK via *Appl*-GAL4 reveals more and larger vacuoles than *loe* alone. **E.** Quantification of the area of vacuoles in *loe* flies with altered levels of Rho kinase (*rok^1^*) shows that increasing ROCK enhances the degeneration whereas reducing ROCK suppresses it. **F.** Quantification showing significantly less vacuolization in *loe* flies in which LIMK is knocked down than in *loe* flies alone. In contrast, overexpressing LIMK significantly increases the area of vacuoles and knocking down LIMK suppresses the degeneration in *loe*. All flies were females and the SEMs and number of brain hemispheres analyzed are indicated. *p<0.05, ***p<0.001. **G.** Overexpressing ROCK in wild type causes degeneration in the retina (arrows) and lamina (arrowheads). re = retina, la = lamina, me = medulla, lo = lobula, lb = lobula plate. Scale bar in **B** = 50 µm (also applies to **C, D**), bar in **G** = 25 µm.

We previously showed that *loe* interferes with isoprenoid synthesis and increases RhoA prenylation [14]. We also showed that decreasing RhoA levels by heterozygosity reduced the vacuolization in *loe*, whereas overexpressing RhoA in neurons enhanced this phenotype. As described above, studies on neuronal outgrowth showed that RhoA activates ROCK which can then phosphorylate LIMK, resulting in the phosphorylation and inactivation of cofilin (Fig. 1A). Therefore we investigated whether the effect of RhoA on *loe*-induced neuronal degeneration is mediated through these downstream targets. To address the role of ROCK, we performed head sections from *loe* control flies and *loe* flies that in addition carried one copy of the recessive lethal *rok^1^* allele and measured the area of vacuoles in each brain hemisphere. Heterozygosity for *rok^1^* reduced the vacuolization to 145.6±20 µm^2^ when measured 5 d after eclosion compared to age-matched *loe* controls with 177.8±11.6 µm^2^ (Fig. 1E), however this difference did not reach statistical significance (p = 0.19). Due to the progressive nature of the *loe* phenotype, we anticipated that a suppressing effect of *rok^1^* could be more prominent in older *loe* flies and therefore also analyzed 10 d old flies. Indeed, we now detected a significant reduction in the area of vacuoles from 388.4±44.7 µm^2^ in *loe* controls (Fig. 1B, E) to 247±33.2 µm^2^ in *rok^1^*/+;*loe/loe* flies (Fig. 1C, E; p<0.05). In contrast, overexpressing ROK, using the pan-neuronal promoter construct *Appl*-GAL4 [25] resulted in a significant increase in vacuole formation in *loe* flies already at 5 d (258±30.2 µm^2^ compared to 177.8±11.6 µm^2^, p<0.05; Fig. 1E). In addition, expression of ROK in the wild type background caused retinal degeneration (arrows, Fig. 1G), similar to the described retinal degeneration when overexpressing RhoA [14]. Although our measurements of vacuolization did not include the retina, these flies showed an increase in vacuole area compared to wild type (p<0.05; Fig. 1E) because we also occasionally found vacuoles in the lamina in these flies (arrowheads, Fig. 1G).

To test whether LIMK plays a role in the neurodegeneration, we overexpressed LIMK in *loe* via *Appl*-GAL4. As expected, overexpression of LIMK significantly aggravated the degenerative phenotype from 177.8±11.6 µm^2^ to 274.2±26.1 µm^2^ in 5 d old *loe* flies (p<0.001, Fig. 1F) and from 388.4±44.7 µm^2^ to 574.7±95 µm^2^ (p<0.05) when aged for 10 d (Fig. 1D, F). Inducing an RNAi construct targeting the limk mRNA had the opposite effect and reduced the area to 97±18.1 µm^2^ (p<0.05, Fig. 1F), confirming an interaction of *loe* with these downstream targets of RhoA. In addition to LIMK, ROCK has also been shown to activate Myosin Light Chain (MLC) kinase and MLC during neuronal outgrowth, which both regulate the contraction of actomyosin [15]. Furthermore, it has been shown by Lee and colleagues [26] that during cell mitosis AMPK can directly phosphorylate MLC. To test whether MLC, the fly orthologue of which is *spaghetti squash*, plays a role in the maintenance of neuronal integrity in *loe*, we used the *sqh^AX3^* mutant line. However, heterozygosity for *sqh^AX3^* did not have an effect on the *loe*-induced degeneration (202.6±13.1 µm^2^ to controls out of the same cross with 190.3±11.1 µm^2^ at 5 d and 383.1±33.0 µm^2^ to controls with 412.8±35.2 µm^2^, Figure S1). Therefore the genetic interactions suggest that the degeneration in *loe* is caused by an increased activation of the LIMK/cofilin/actin pathway and not through effects on actomyosin.

### *Loe* shows Increased Levels of Phospho-cofilin

**Figure 2:**
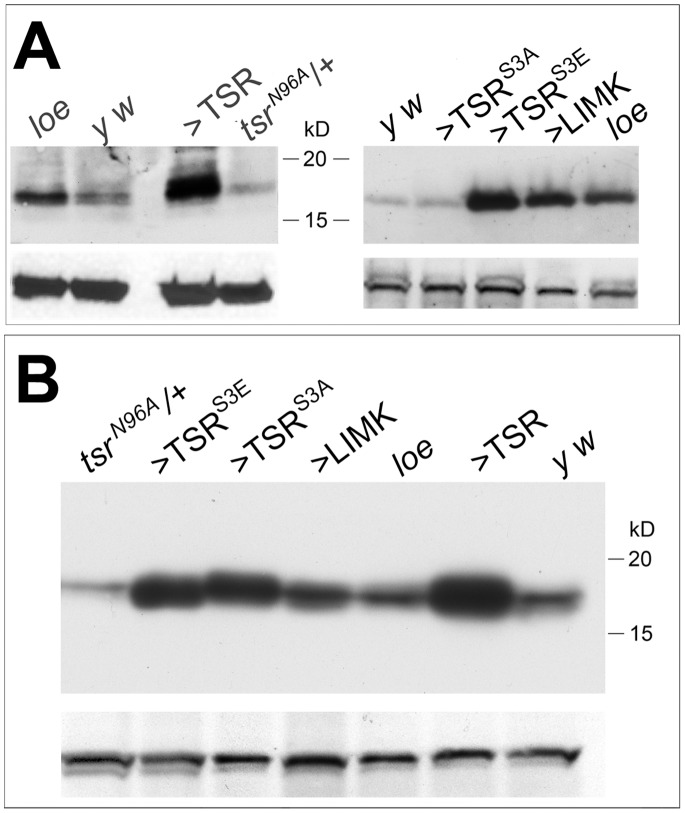
*loe* shows increased levels of p-cofilin. A. A Western Blot using α-p-cofilin reveals an increase in p-cofilin in *loe* compared to *y w* control flies, the genetic background of *loe*. Overexpressing TSR, TSR^S3E^, or LIMK in neurons via *Appl*-GAL4 also increases the levels of p-cofilin whereas *tsr^N96^* ^***A***^/+ flies show reduced levels. Expression of TSR^S3A^ did not affect p-cofilin levels. Head extracts from 10 flies were loaded per lane. B. A Western blot using α- cofilin shows an increase in total cofilin in flies expressing TSR, TSR^S3E^, or TSR^S3A^ with *Appl*-GAL4. *tsr^N96A^*/+ flies show a decrease in total cofilin whereas LIMK overexpressing flies (note the higher load in this lane) and *loe* flies do not show a change in total cofilin levels. Head extracts from 5 flies were loaded per lane. Loading controls using α-tubulin are shown below each blot.

As pictured in figure 1A, LIMK phosphorylates cofilin, which in *Drosophila* is encoded by the *twinstar (tsr)* gene, and this has been shown to inactivate cofilin [18]. Dephosphorylated by the phosphatase Slingshot (SSH), cofilin promotes actin depolymerization and thus formation of globular actin (G-actin). Because *loe* activates the RhoA/LIMK pathway and therefore cofilin phosphorylation, *loe* mutant flies should show an increased level of p-cofilin. Performing Western blots with an anti-p-cofilin antibody, indeed confirmed an increase in p-cofilin in *loe* head extracts compared to *y w* control flies, the genetic background of *loe* (Fig. 2A). We also included TSR overexpressing and heterozygous *tsr^N96A^* mutant flies which showed the expected increase or decrease in p-cofilin, respectively (Fig. 2A). In addition, we tested whether expression of a constitutively active form of TSR (TSR^S3A^; [19]) affected the levels of p-cofilin and as expected this was not the case (Fig. 2A). In contrast, expression of the phosphomimetic TSR^S3E^ [19] dramatically increased p-cofilin levels, as did expression of LIMK (Fig. 2A). To determine whether the *loe*-induced changes in cofilin are specific for the phosphorylated form, we also performed Western blots with an antibody recognizing total cofilin. As shown in figure 2B, this revealed no increase in total cofilin in *loe* showing that *loe* indeed specifically increases the levels of p-cofilin. As expected, total cofilin levels were decreased in the heterozygous *tsr^N96A^* mutant and increased in all lines that overexpressed TSR (TSR, TSR^S3E^, or TSR^S3A^). Although the levels of total cofilin were slightly increased in LIMK expressing flies in this blot this is due to an increase in the load in this lane because when loading equal amounts we did not detect an increase (Figure S2).

### Reducing Cofilin Levels Improves *Loe*-induced Phenotypes

**Figure 3:**
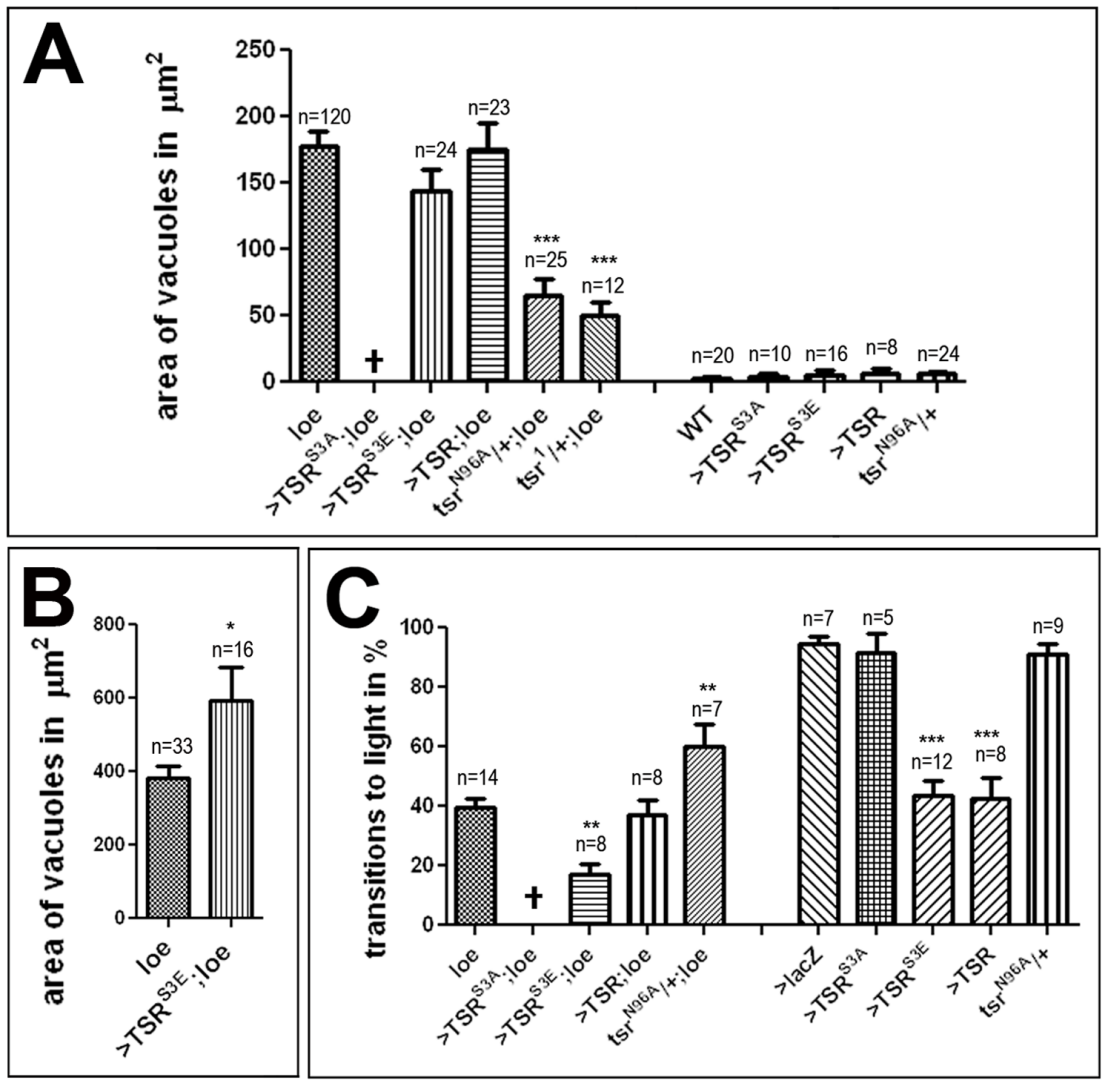
*loe* interactions with *tsr*. A. 5*loe* flies heterozygous for *tsr^N96A^* or *tsr^1^* show a significantly reduced area of vacuoles compared to *loe* alone. Overexpression of TSR or phosphomimetic TSR (TSR^S3E^) via *Appl*-GAL4 has no effect on vacuole formation in 5 d old *loe* flies. Combining the constitutively active TSR^S3A^ construct with *loe* is developmental lethal and could not be tested. Neither overexpression of TSR, TSR^S3A^, TSR^S3E^ nor removing one copy of *tsr* caused vacuole formation in the wild type background. B. Analyzing *loe* flies expressing TSR^S3E^ at 10 d of age reveals a significant increase in vacuolization compared to *loe* controls. C. *tsr^N96A^*/+;*loe/loe* flies show increased performance in fast phototaxis experiments compared to *loe* when tested at 5 d of age. Expression of the phosphomimetic TSR^S3E^ construct with *Appl*-GAL4 enhances the locomotion deficits of *loe* and also has a significant effect when induced in the wild type background. Expression of TSR^S3A^ or heterozygosity for *tsr^N96A^* had no effect in wild type but expression of TSR also causes behavioral deficits. A, B. n = number of brain hemispheres analyzed are indicated in each graph. C. n = number of independent experiments with groups of 3–6 flies. The error bars indicate SEMs. All the analyzed flies were females. *p<0.05, **p<0.01, ***p<0.001.

If the loss of active cofilin plays a role in *loe-*induced phenotypes, expression of the constitutively active form TSR^S3A^ should be beneficial. We therefore tried to generate *loe* flies that expressed TSR^S3A^ via *Appl*-GAL4, however we were not able to get adult flies of this genotype. To determine whether this could be due to a deleterious effect of TSR^S3A^ overexpression independent from the *loe* mutant, we expressed TSR^S3A^ in the wild type background. This cross produced viable adults which had neither a degenerative (Fig. 3A) nor a behavioral phenotype in fast phototaxis assays (Fig. 3C) when 5 d old. However, we were also not able to obtain *loe* homozygous flies that carried only the TSR^S3A^ construct without the driver, suggesting that, though there is an interaction with *loe*, this is caused by effects independent from TSR^S3A^ expression (possibly effects caused by the insertions). Next we tested whether it might be the increase in p-cofilin that is actually deleterious and not the decrease in active cofilin. To address this issue, we expressed the phosphomimetic TSR^S3E^ construct in *loe* and although this had no consequences for the degenerative phenotype at 5 d of age (with 144±16.1 µm^2^, Fig. 3A), it did result in an increase in the area of vacuoles when the flies were aged for 10 d (Fig. 3B). In addition, expression of TSR^S3E^ aggravated the locomotion deficits of *loe*, decreasing the percentage of successful transitions from 39.4±3.4% to 17±3.6% (p<0.01, Fig. 3C). Moreover, expression of this construct alone via *Appl*-GAL4 caused a significant reduction in the performance compared to wild type (43.7±4.9% versus 94.5±2.3%; p<0.001; Fig. 3C). We also included expression of the wild type TSR construct, which did not significantly affect the phenotypes of *loe* but also significantly decreased the performance in the phototaxis assay in the wild type background (Fig. 3C). Finally, we tested *loe* flies that were heterozygous for the lethal *tsr^N96A^* allele which, as shown in figure 3A and 3C, significantly improved the degenerative phenotype (to 65±12.3 µm^2^; p<0.001) as well as the locomotion deficits (to 60±7.8%; p<0.01) but had no effect in the wild-type background. The suppressing effect of *tsr^N96A^* was confirmed with a second allele, *tsr^1^*, though we only tested this combination for the degenerative phenotype (Fig. 3A). As shown in figure 2A, heterozygosity for *tsr* did reduce the amount of p-cofilin and this might mediate the beneficial effect of lacking on copy of *tsr*.

**Figure 4:**
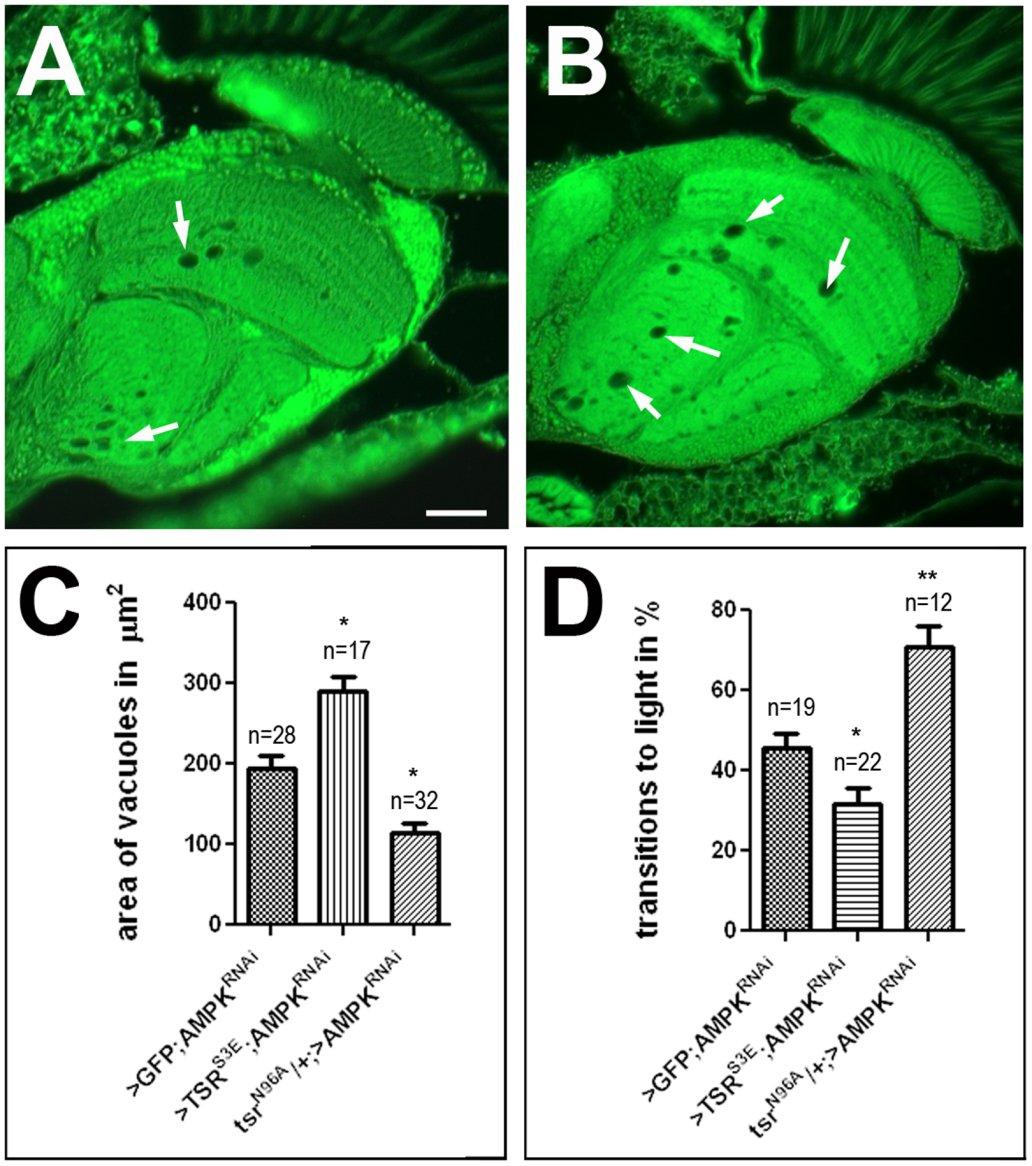
TSR also interacts with the AMPK knockdown. A. A 9*elav*-GAL4, *Appl*-GAL4;UAS-AMPKγ RNAi fly also reveals formation of spongiform lesions (arrows). B. This phenotype is enhanced when expressing TSR^S3E^ in these flies. C. Heterozygosity for *tsr^N96A^* suppresses the degeneration in the pan-neuronal knockdown of 9 d old AMPKγ via *elav*-GAL4, *Appl*-GAL4 whereas co-expression of TSR^S3E^ significantly enhanced vacuole formation. UAS-GFP was expressed in the control AMPKγ knockdown to have equal numbers of UAS constructs. D. A comparable suppressing and enhancing effect of *tsr^N96A^* and TSR^S3E^ is detectable when using 9 d *elav*-GAL4, *Appl*-GAL4;UAS-AMPKγ RNAi flies and performing fast phototaxis assays. Bar in A = 25 µm and also applies to B.

To confirm the interactions observed with the *loe* allele, we also used a knockdown approach with an RNAi construct against all AMPKγ transcripts We previously showed that expression of this construct with *elav*-GAL4 alone did not induce detectable degeneration, although it did cause locomotion deficits [14]. However, combining the pan-neuronal driver *elav*-GAL4 with *Appl*-GAL4 and aging them for 9d, these knockdown flies showed a degeneration similar to the one observed in *loe* with a vacuole area of 194.1±15.6 µm^2^ (Fig. 4A, C). Expression of the phophomimetic TSR^S3E^ significantly increased this phenotype (289.4±17.8 µm^2^; p<0.05, Fig. 4B, C) whereas removing one copy of *tsr* (*tsr^N96A^*) suppressed the degenerative phenotype to 114.7±12.1 µm^2^ (p<0.05). Similarly, the behavioral phenotype of the knockdown was enhanced by co-expression of TSR^S3E^ and suppressed by heterozygosity for *tsr^N96A^* (Fig. 4D). Together these results suggest that an increase in p-cofilin plays a role in the progressive neurodegeneration and behavioral deficits observed in the *loe* mutant.

### Reducing SSH Levels Ameliorates *Loe*-induced Phenotypes

**Figure 5:**
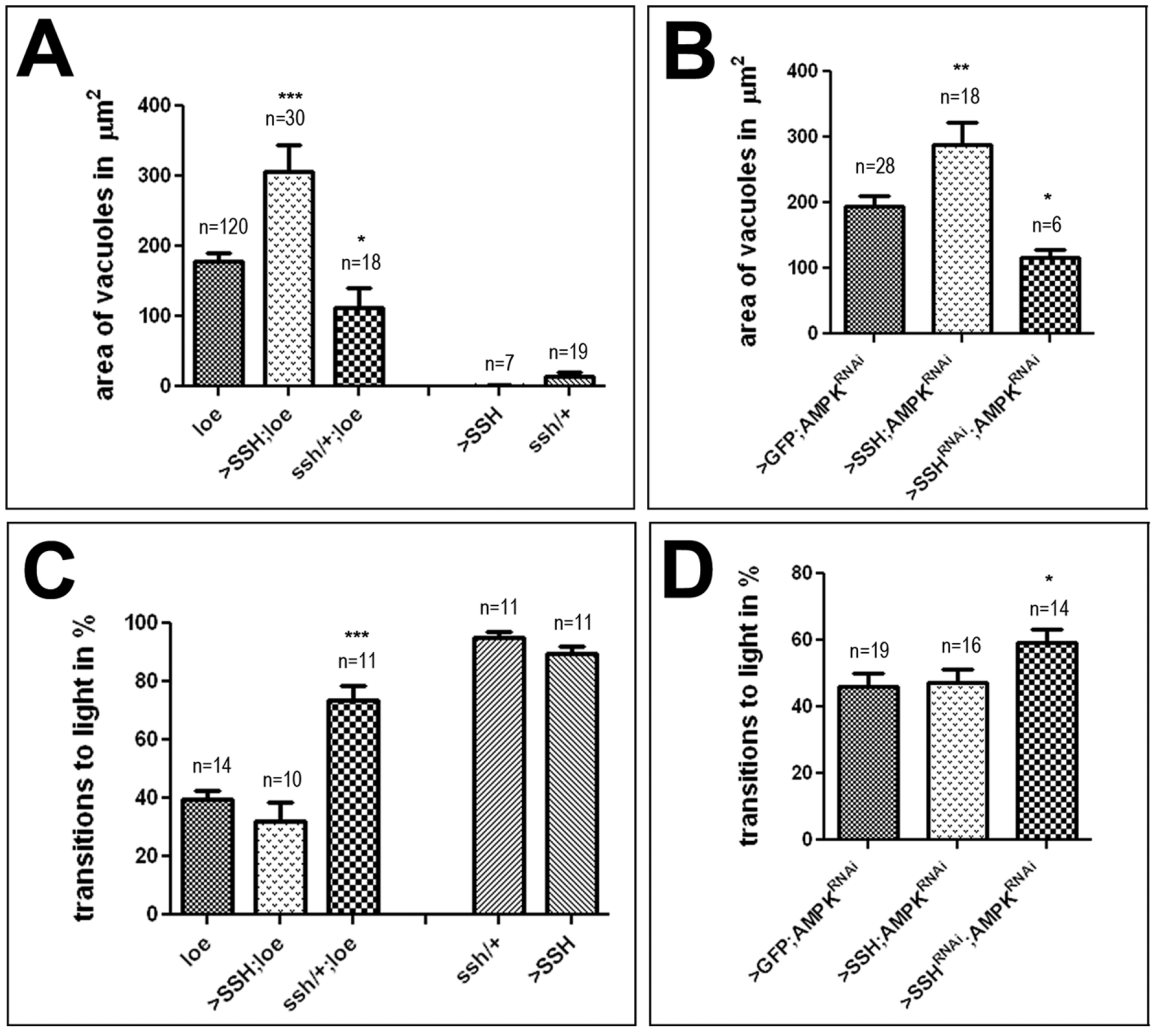
Reducing SSH levels suppresses *loe* phenotypes. ***A.*** *loe* flies expressing additional SSH via *Appl*-GAL4 show an enhancement of vacuole formation whereas heterozygosity for *ssh^1–63^* reduces vacuoles formation at 5 d of age. Neither an increase nor a decrease of SSH levels alone caused degeneration. B. Similarly, reducing SSH levels by co-expressing an SSH-RNAi construct with the AMPKγ RNAi construct via *Appl*-GAL4, *elav*-GAL4 decreases vacuole formation in 9 d old flies when compared to controls expressing GFP with the AMPKγ RNAi construct. In contrast, co-expression of SSH significantly enhances vacuole formation. **C.** Heterozygosity for *ssh^1–63^* also suppresses the behavioral phenotype of 5 d old *loe* flies in fast phototaxis experiments, whereas overexpression of SSH (with *Appl*-GAL4) did not change the performance of *loe*. *ssh^1–63^*/+ or SSH overexpression flies alone did not show locomotion deficits. D. An improvement in performance is detectable in 9 d old flies when the AMPKγ RNAi construct is co-expressed with a SSH-RNAi construct (using *Appl*-GAL4, *elav*-GAL4). A, B. n = number of brain hemispheres analyzed are indicated in each graph. **C, D.** n = number of independent experiments with groups of 3–6 flies. All flies were females and the SEMs are indicated. *p<0.05, **p<0.01, ***p<0.001.

**Figure 6:**
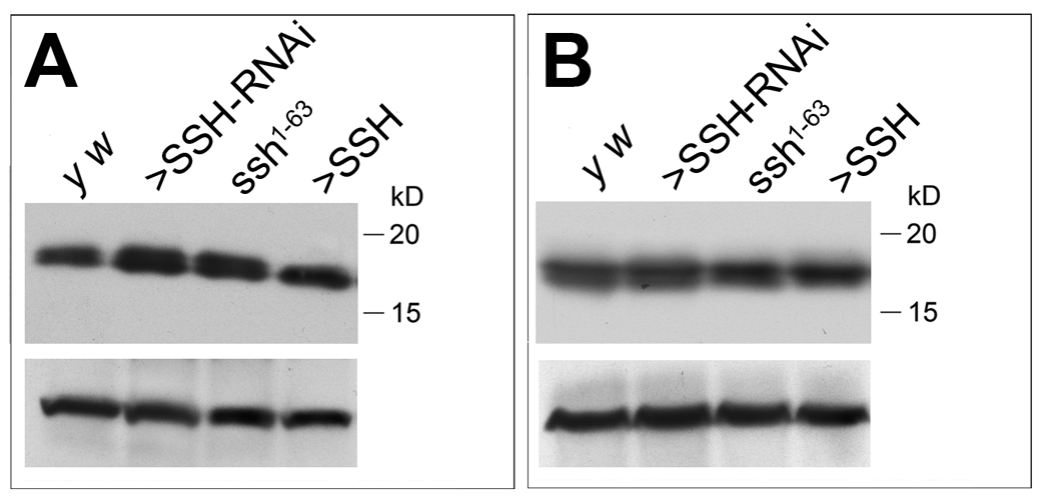
Overexpression of SSH does not reduce p-cofilin. A. A Western blot using α-p-cofilin shows an increase in flies with reduced SSH levels. Overexpressing SSH does not reduce p-cofilin compared to wild type but seems to slightly increase the levels. Lysates from 10 heads were loaded in each lane. B. The levels of total cofilin were not altered when manipulating SSH. Lysates from 5 heads were loaded in each lane.

During neuronal outgrowth, cofilin is activated by dephosphorylation via the protein phosphatase Slingshot (SSH). We therefore also analyzed whether *loe* genetically interacts with SSH. We first tested the effects of elevated SSH levels, via *Appl*-GAL4, which increased the vacuole area in 5 d old *loe* mutants from 177.8±11.6 µm^2^ to 307.3±37 µm^2^ (Fig. 5A; p<0.001) but had no significant effect on the behavioral deficits of *loe* in the fast phototaxis assay (Fig. 5C). Reducing SSH levels by heterozygosity for the lethal *ssh^1–63^* allele suppressed both, the degenerative and behavioral phenotype of *loe* with a vacuole area of 112.9±29.3 µm^2^ and a performance index of 73.8±7.8% (p<0.05 and p<0.001). Neither overexpression nor reduction of SSH in an otherwise wild-type background had an effect on brain morphology or locomotion on its own (Fig. 5A, C). Repeating these experiments with the 9 d old AMPKγ knockdown flies (via *Appl*-GAL4, *elav*-GAL4), we again detected a significant enhancement of the degenerative phenotype when additional SSH was expressed (194.1±15.6 to 288.3±34.4 µm^2^, p<0.01) and a also suppression when SSH levels were reduced by co-induction of a SSH RNAi construct (to 116.3±11.5 µm^2^, p<0.05; Fig. 5B). Concerning the behavioral deficits, overexpression of SSH had no effect, whereas reducing SSH suppressed the phenotype in the fast phototaxis assay (46.2±3.7% versus 59.5±4%; p<0.05; Fig. 5D). These results further support our observations with TSR that the *loe*/AMPKγ-induced phenotypes appear not to be due to the loss of unphosphorylated, active cofilin. Nevertheless these results were surprising because SSH can also dephosphorylate and inactivate LIMK [27] and SSH overexpression should consequently also result in a reduction in p-cofilin. To verify that altering SSH levels had the expected effects on cofilin and p-cofilin, we again performed Western blots. As predicted by SSH’s role as a cofilin phosphatase, reducing SSH levels by heterozygosity for *ssh^1–63^* or by inducing the RNAi construct via *Appl*-GAL4 indeed resulted in an increase in p-cofilin (Fig. 6A). However, increasing SSH levels did not reduce p-cofilin levels but might even slightly increase them (Fig. 6A). Neither manipulation had a detectable effect on total cofilin (Fig. 6B). One would therefore expect that heterozygosity for *ssh* would enhance the phenotypes but as we showed in figure 5, we observed the opposite effect, indicating that SSH might have additional functions besides its role in dephosphorylating cofilin. In contrast to our expectation that overexpression of SSH would reduce p-cofilin it slightly increased the levels, correlating with the enhancement of the *loe* phenotypes. Interestingly, in addition to the well described function of SSH during neuronal outgrowth in promoting F-actin severing via its effects on cofilin and LIMK, *in vitro* experiments have suggested that SSH can also have the opposite function and stabilize F-actin [28].

### *Loe* Mutants have Increased Levels of Filamentous Actin

**Figure 7:**
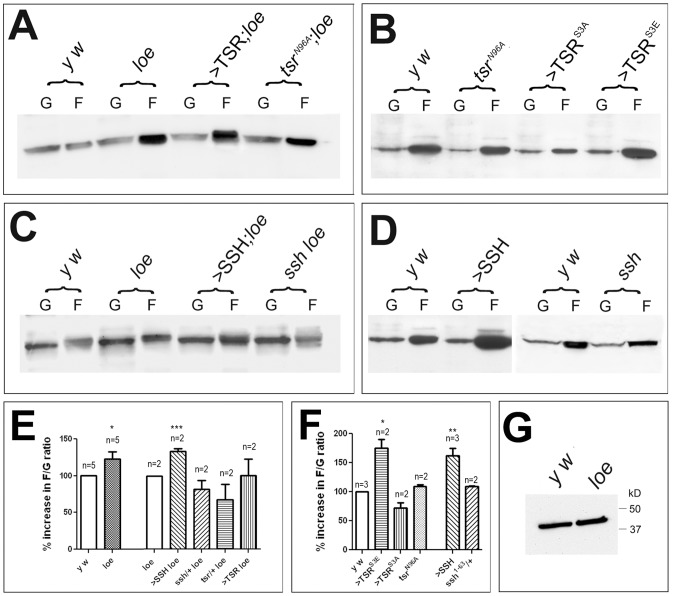
Filamentous actin is increased in *loe*. **A.** *loe* flies show an increase in filamentous (F) actin compared to *y w* controls (G = globular actin). This is not altered by expressing TSR but heterozygosity for *tsr^N96A^* reduces F-actin in *loe* (compare lane 4 and 8). **B.** Expressing TSR^S3E^ via *Appl*-GAL4 increases F-actin whereas flies expressing TSR^S3A^ show less F-actin. **C.** Expressing additional SSH in *loe* further increases F-actin (compare lane 4 and 6) whereas *ssh^1–63^*/+;*loe/loe* flies show a reduction in F-actin (compare lane 4 and 8). **D.** Induction of SSH increases F-actin levels whereas heterozygosity for *ssh^1–63^* had no effect. **E.** Quantification of the F-actin to G-actin ratio normalized to controls reveals a significant increase in *loe* compared to *y w* controls and in *loe* flies overexpressing SSH to *loe*. **F.** The ratio of F-actin to G-actin is also significantly increased in flies expressing TSR^S3E^ or SSH. The number of independent experiments and the SEMs are indicated. *p<0.05, **p<0.01, ***p<0.001. **G.** The levels of total actin appear unchanged in *loe*.

To directly study the effects of *loe* on actin polymerization, we measured the levels of filamentous versus globular actin (Fig. 7A, B). Due to changes in the ratio of F-actin/G-actin in different preparations, we normalized the ratio in *loe* to the ratio of *y w* controls from the same preparation. Performing five independent Western blots we found a 23.2±9.8% increase in the ratio of F-actin/G-actin in *loe* (Fig. 7E). In contrast, we did not detect changes in the levels of total actin between control and *loe* flies (Fig. 7G). Next, we tested whether manipulations of TSR and SSH can affect this ratio in *loe* and in agreement with the suppression of the degenerative and behavioral phenotypes by heterozygosity for *tsr^N96A^*, the ratio was shifted towards less F-actin in *tsr^N96A^*/+;*loe/loe* flies with a ratio of 67.9±20.8% although it did not reach statistical significance with the two blots analyzed (Fig. 7A, E). TSR overexpression did not seem to have an effect (101.0±22.1%). Heterozygosity for *tsr^N96A^* in a wild type background had no effect on the F-actin/G-actin ratio but as expected expression of TSR^S3E^ significantly increased F-actin (Fig. 7B, F). Expression of TSR^S3A^ reduced F-actin but this did not reach statistical significance. Extending this analysis to SSH, we found a statistically significant increase in F-actin when additional SSH was expressed in *loe* compared to *loe* alone (134.0±3.4%) and a decrease (not reaching significance) when heterozygous for *ssh^1–63^* (82.1±12.2%; Fig. 7C, E), consistent with the genetic interactions. Further supporting our hypothesis that SSH promotes F-actin stabilization, we also found a significant increase in F-actin when expressing SSH in the wild type background (162.3±13.9%; Fig. 7D, F). Heterozygosity for *ssh^1–63^* had no effect. Finally, we tested the effects of LIMK expression on F-actin levels and in agreement with its function in increasing p-cofilin it increased F-actin levels (Figure S3). Together our results that increasing SSH levels switches the ration towards more F-actin in the *loe* as well as wild type background strongly support a stabilizing function of SSH, as described previously in *in vitro* assays [28].

**Figure 8:**
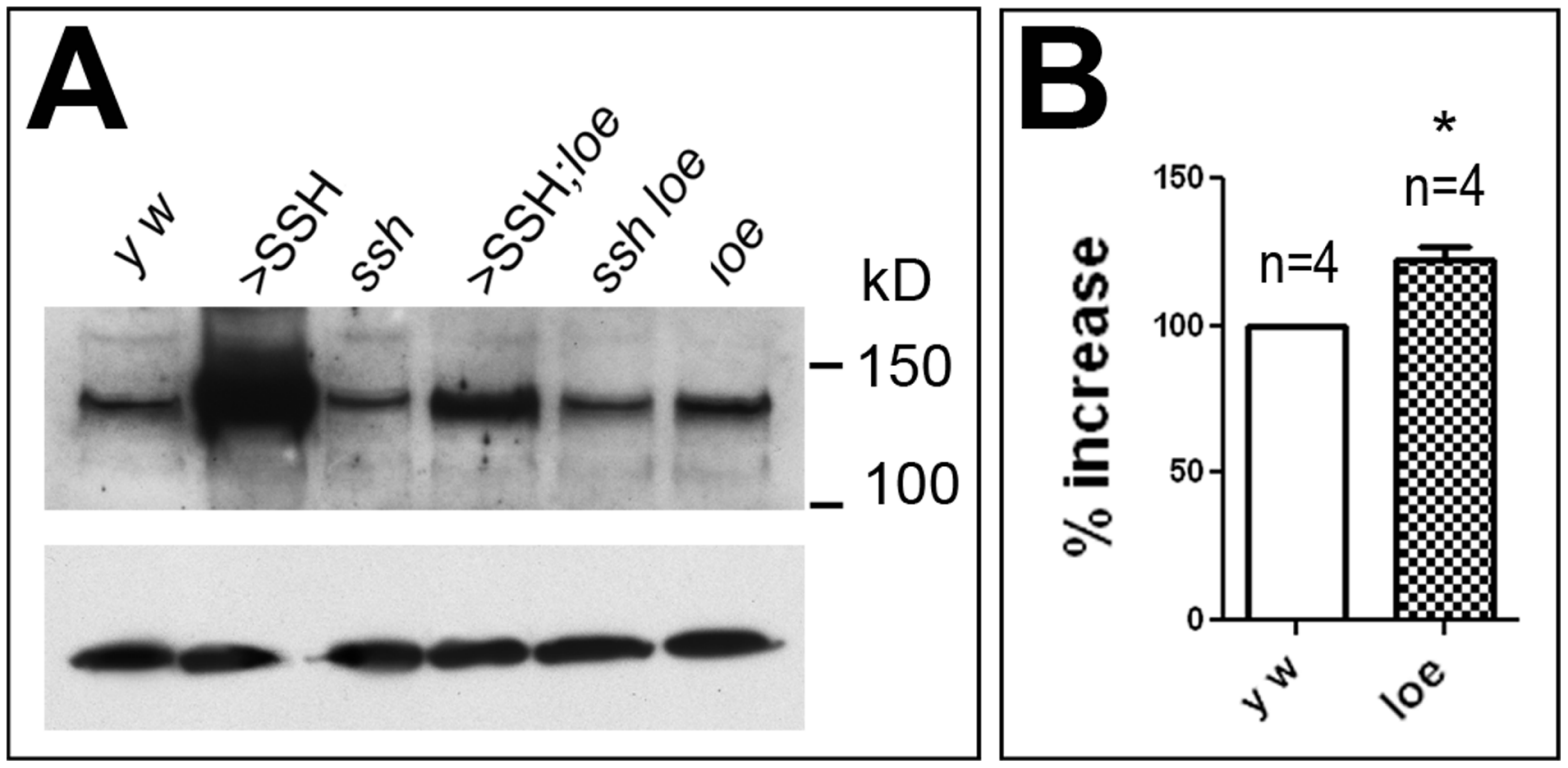
SSH levels are increased in *loe*. **A.** A Western blot using α-SSH shows increased levels of SSH in *loe* and in SSH overexpressing flies whereas the levels are reduced in *ssh^1–63^*/+ flies. Expressing SSH in the *loe* background results in a weaker band when compared to expression in the wild type background. A loading control using α-tubulin is shown below. **B.** Quantification showing the increase in SSH in *loe* compared to controls. The number of independent experiments and the SEMs are indicated. *p<0.05.

Finally, we performed Western blots to determine whether the excess in F-actin may be stabilized by additional SSH and as shown in figure 8A and B, the increase in F-actin in *loe* flies indeed correlates with a 22.0±5.1% increase in SSH levels. Confirming our manipulations of SSH, induction of SSH in wild type dramatically increased SSH levels and using *ssh^1–63^*/+ flies reduced them. Surprisingly however, expressing SSH in *loe* flies seemed to reduce the amount of additional SSH when compared to the expression in wild type (Fig. 8A), indicating that AMPK may regulate SSH by an unknown mechanism.

### *Loe* Interferes with Neuronal Maintenance

**Figure 9:**
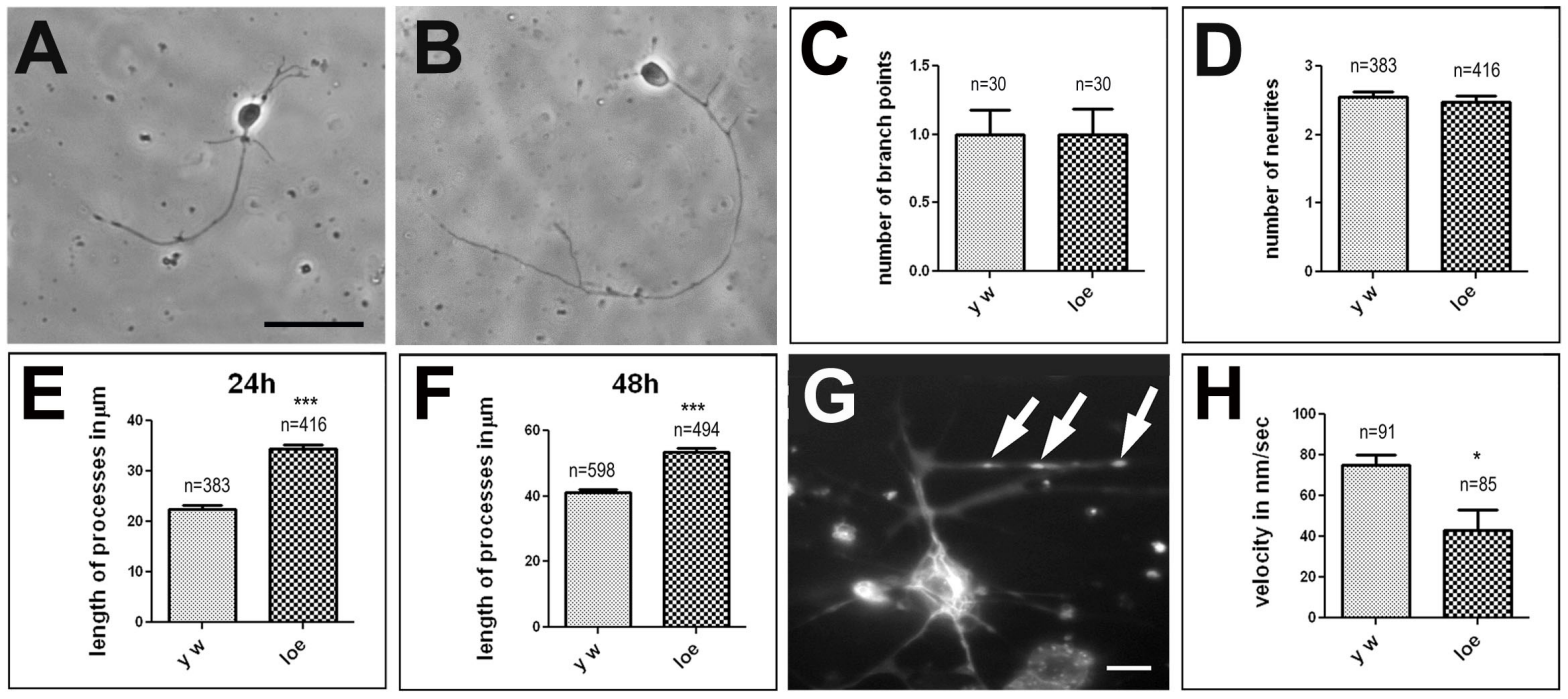
*loe* affects neurite length and axonal transport in cultured neurons. **A.** *y w* control neuron 24 h in culture. **B.** *loe* neuron 24 h in culture. **C.** Counting the number of branch points does not reveal a difference between *loe* neurons and controls. **D.** Counting the number of neurites does not reveal a difference between *loe* neurons and controls. **E.** The average length of the longest neurite in primary neuronal cultures is significantly increased in *loe* neurons cultured for 24 h compared to *y w* control neurons. **F.** In contrast, *loe* neurons show shorter neurites when cultured for 48 h. **G.** A cultured neuron labeled with Mitotracker to follow the movement of mitochondria (arrows). **H.** The average velocity of mitochondria transported along neurites is significantly decreased in *loe* mutant neurons. The number of analyzed neurons and the SEMs are indicated. Scale bar in **A** = 10 µm (also applies to **B**), scale bar in **G** = 2 µm. *p<0.05, ***p<0.001.

Actin dynamics have been shown to play a crucial role during neuronal outgrowth and although *loe* mutant flies do not exhibit detectable defects in the development of the brain [9], we investigated whether we could detect more subtle effects using primary neuronal cell cultures. Whereas we did not find changes in neurite numbers or branching patterns between control and *loe* neurons grown for 24 h (Fig. 9C, D), measurements of the longest neurite of each cell revealed a significant increase in length in *loe* (Fig. 9B) compared to controls (Fig. 9A) with 22.4±0.8 µm in wild type and 34.4±0.9 µm in *loe* (p<0.001; Fig. 9E). However after 48 h in culture, *loe* neurons were actually significantly shorter with 41.1±1 µm compared to 53.4±1.3 µm in the control (p<0.001; Fig. 9F). This result suggests that the increased activation of the RhoA/LIMK pathway and the resulting increase in p-cofilin and F-actin does not prevent neurite outgrowth but initially even promotes it. However, whereas wild-type neurons continue growing, *loe* neurons either appear stop growing or cannot maintain their neurites. To determine whether *loe*-associated changes in actin dynamics can interfere with cellular processes that might mediate the stalling in growth, we analyzed axonal transport in these cultured neurons. Using Mitotracker, we determined the velocity of mitochondrial transport along neurites in neurons cultured for 24 h (Fig. 9G) and found that it is significantly reduced in *loe* cells compared to wild type controls (p<0.01; Fig. 9H). This defect in axonal trafficking could eventually deplete neurites of factors they need to maintain their integrity or to promote further growth.

**Figure 10:**
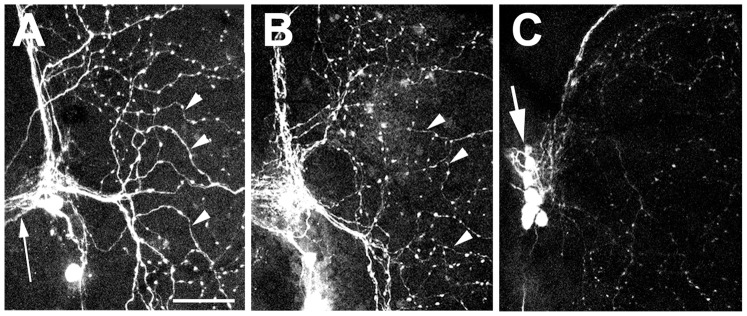
*loe* flies show progressive neuritic degeneration. **A.** Expression of GFP via *pdf*-GAL4 reveals the pattern of the PDF neurons and their projections into the medulla (arrowheads) in a 5 d old control fly. **B.** In a 1 d old *loe* fly, the overall pattern appears quite normal although the staining of the projections into the medulla appears somewhat weaker. **C.** In a 5 d old *loe* fly the arborizations in the medulla are thin or missing whereas the cell bodies are still easily detectable (arrow). Scale bar in **A** = 10 µm also applies to **B** and **C**).

To investigate whether defects in neurites are also the initial event in the neuronal degeneration observed in the *loe* mutant, we marked a small group of neurons in the adult brain by expressing GFP with *pdf*-GAL4. This promoter construct is derived from the gene for the pigment dispersing factor (PDF) which is expressed in a few clusters of cells, including a cluster of eight cells localized at the anterior base of the medulla called [16]. Four of these neurons send arborizations in the medulla forming a network that covers the entire surface (arrowheads, Fig. 10A) whereas their axons project to the contralateral medulla via the posterior optic tract (arrow). As seen in figure 10B, these neurons are present and have extended their axons and dendrites in a 1 d old *loe* fly, although the staining in the medulla appeared less intense (arrowheads). In contrast, the axonal and dendritic staining was very weak or in some areas missing in 5 d old *loe* flies although the cell bodies were still present (arrow, Fig. 10C). This suggests that neuronal outgrowth proceeds normal in *loe* but the neurons cannot maintain their neuritic network.

## Discussion

We previously showed that *loe* mutant flies show increased prenylation and genetically interact with RhoA, whereby reducing RhoA levels suppressed *loe*-induced phenotypes [14]. Rho GTPases are key regulators of the actin network through their downstream targets PAK and ROCK [17]. Because RhoA mostly signals through ROCK, whereas PAKs are downstream targets of Rac and Cdc42, we tested whether ROCK can affect the neurodegenerative defects in *loe*. Indeed, mutations in *rok* suppressed the *loe*-induced degeneration whereas additional ROCK expression increased it. In addition induction of ROCK in wild type also caused a degenerative phenotype although it was mostly restricted to the retina and lamina. This may indicating that photoreceptors may be more sensitive to increased ROCK expression and due to LOE not being detectable in the retina, hyperactivation of ROCK via *loe* would not affect the eye. ROCK can activate two downstream kinases, MLC kinase, which through MLC regulates actin fiber contraction, and LIM kinase, which regulates actin polymerization via cofilin [15]–[17], [29]. Performing genetic interaction tests showed that *loe* interferes with the latter pathway, whereas mutations in MLC had no effect on the *loe* phenotype. During neuronal outgrowth, LIMK has been shown to phosphorylate and inactivate cofilin, thereby inhibiting the actin severing activity of cofilin. Consistent with this role of LIMK and its hyperactivation by *loe*, we found an increase in p-cofilin levels in *loe* without an increase in total cofilin levels and an increase in filamentous actin. We therefore hypothesized that the loss of unphosphorylated, active cofilin is responsible for the deleterious phenotypes in *loe*. Unfortunately we could not investigate effects of the constitutively active TSR^S3A^ on *loe* because even without a promoter construct the combination of *loe* and the TSR^S3A^ construct resulted in developmental lethality. In contrast, expression of a phosphomimetic form of TSR (TSR^S3E^) in *loe* did not cause lethality but enhanced the locomotion deficits and degenerative phenotype in *loe* and in the AMPKγ knockdown. Although p-cofilin is mostly considered to be an inactive form with no biological function, our results show that its accumulation can have deleterious consequences and not only in combination with mutants in AMPKγ but also in a wild type background because its expression via *Appl*-GAL4 caused significant deficits in the fast phototaxis assay (although no detectable degeneration). An active role of p-cofilin in the phenotypes observed after the loss of AMPKγ is further supported by the suppression of the degeneration and behavioral deficits when *loe* or the knockdown was heterozygous for loss-of function mutations in *tsr* because, as shown in figure 2, heterozygosity for *tsr* reduced p-cofilin levels. Furthermore, these experiments support our assumption that it is not the loss of active cofilin that induces the deleterious effects of *loe* because heterozygosity for *tsr* also lowers the levels of unphosphorylated cofilin. Although not many studies have focused on p-cofilin, a possible negative effect of increased p-cofilin levels has been suggested by experiments in cell culture. Siudeja and colleagues recently showed that Pantothenate kinase deficient cells showed an increase in p-cofilin and a reduced ability to form neurites [30]. In addition, treatment of hippocampal neurons with Aβ increased the phosphorylation of cofilin and resulted in changes in the actin cytoskeleton, neuritic dystrophy, and cell death [23], [31]. Though independent of actin, p-cofilin has also been described to have an active function in regulating phospholipase D1 [32]. However, because we could not study the effects of TSR^S3A^, it is possible that also changes in active cofilin or effects on the ratio of active cofilin to p-cofilin play a role in the *loe* phenotype.

p-cofilin is activated by dephosphorylation through the SSH phosphatase, which can also dephosphorylate and inactivate LIMK, therefore SSH is promoting F-actin severing in two ways [27], [33], [34]. Due to this dual function of SSH, its overexpression should counterbalance the hyperactivation of LIMK and the increase in p-cofilin and suppress the deleterious phenotypes even if the loss of active cofilin does not play a role in *loe*. However, we observed the opposite effect with additional SSH expression enhancing the vacuole formation in *loe* as well as in the AMPKγ knockdown (but it had no effect on the behavioral phenotype). Also, overexpression of SSH did not reduce p-cofilin. In contrast, reducing SSH levels, either by a mutation or by an RNAi-mediated knockdown, suppressed the degenerative and behavioral phenotype, although it did increase p-cofilin, suggesting that the interaction between *loe* and SSH is quite complex. This is also indicated by our result that the levels of SSH after overexpression are reduced in the *loe* background when compared to overexpression in the wild type background. Interestingly, SSH can directly bind F-actin [35] and human SSH1 has been shown to promote F-actin stabilization and bundling *in vitro* [28]. Although F-actin binding can activate the phosphatase function of SSH [36], the F-actin stabilizing function is independent of the phosphatase activity and SSH1 expression can protect from cofilin-induced de-polymerization [28]. Because loss of SSH does increase p-cofilin but suppresses the phenotypes, it is likely that the interaction between *loe* and SSH is independent form SSH’s phosphatase activity. In agreement with an involvement of the F-actin stabilization function, additional expression of SSH increased F-actin levels in wild type and, correlating with its enhancing function also in *loe.* Although it did not reach statistical significance, reducing SSH decreased F-actin showing the right tendency. Therefore our results suggest that in contrast to the well-established role of SSH in promoting actin severing during neuronal outgrowth, its major function in the mature nervous system may be F-actin stabilization. That SSH is necessary as a stabilizing factor could also explain why the increase in p-cofilin in *ssh* mutants and the knockdown does not have an enhancing effect and does not increase F-actin, because although more p-cofilin is made the F-actin can then not be stabilized.

That *loe* does not dramatically disrupt neuronal outgrowth is shown by the normal appearance of the nervous system in first instar larvae and newly eclosed flies [10] and by the quite normal pattern of projections of PDF neurons when 1 d old. Furthermore our primary neuronal cell culture studies show that the neurons can initiate neuronal outgrowth and grow even fast than the controls during the first 24 h. Although activation of RhoA is generally associated with growth cone collapse and retraction during development [15], [17] it can also promote outgrowth, probably by inhibiting the severing activity of cofilin thereby resulting in increased stabilization of novel actin filaments [15], [37], [38]. Therefore the increase in F-actin in *loe* may enable them to grow faster. However, these changes in the cytoskeleton also have deleterious consequences even at this stage because mitochondrial transport was already reduced in neurons cultured for 24 h. Whether the effect on mitochondria, which are transported along microtubule, is caused by a disorganization of the cytoskeleton due to the changes in actin filaments or a more direct effect still has to be determined. At least in endothelial cells, LIMK activity has been shown to play a role in microtubule stability [39] and therefore *loe* could have a more direct effect on the microtubule network and mitochondrial transport. Due the normal development of the brain of *loe* flies, neuronal outgrowth seems not to be disrupted in vivo and could possibly even occur faster. However, as the staining of the PDF neurons in 5 d old flies reveals, the neuritic network cannot be maintained. Similarly, the primary neurons derived from *loe* are not able to continue the increased growth rate of their neurites which eventually become shorter than in controls. This inability to maintain their neuritic network may be caused by the decrease in axonal transport we observed, however changes in actin dynamics affect many cellular processes that when disturbed may interfere with neuronal integrity. Together our results show that changes in the RhoA/LIMK pathway and actin dynamics are an important factor in the neurodegenerative phenotype in *loe*, although the interactions between *loe* and actin dynamics may be quite complex. In addition, due to the role of AMPK in a variety of pathways, we expect that *loe* has pleiotropic effects and therefore we do not expect that the changes in actin dynamics are the only cause of the phenotype. That effects of *loe* on other pathways do play a role in the neurodegeneration that we observe in this mutant is also suggested by the result that TSR^S3E^ and SSH expression can enhance *loe*-induced neurodegeneration but do not cause neurodegeneration in the wild type background although they show a strong increase in F-actin. Nevertheless, our results show that the RhoA pathway and actin dynamics play an important role in the maintenance of the mature brain. However, some of the components seem to have different functions in the adult nervous system than during development with p-cofilin possibly playing a more active role instead of just being an inactive form and SSH acting as a stabilization factor for actin filaments instead of promoting actin severing.

## Materials and Methods

### Fly Stocks

All fly stocks were maintained and raised under standard conditions at 25°C. The *Appl*-GAL4 line was kindly provided by L. Torroya (Universidad Autonoma de Madrid, Spain). The *loe* mutation is described in [9]. *elav*-GAL4, *rok^1^*, *tsr^1^, tsr^N96A^,* UAS-*tsr*, UAS-*tsr^S3A^*, UAS-*tsr^S3E^*, UAS-*limk*, UAS-*ssh*, *ssh^1–63^* and UAS-SNF4Aγ RNAi (JF02060) were obtained from the Bloomington stock center. The UAS-SSH RNAi line (GD11869) was acquired from the Vienna Drosophila RNAi center.

### Microscopy and Measurement of Vacuolar Pathology

Paraffin sections were performed as described in [40]. Briefly, flies were fixed in Carnoy’s fixative for 4 h, washed and dehydrated in absolute ethanol and incubated in methylbenzoate overnight. After a 1 h incubation in 1∶1 methylbenzoate/paraffin, followed by several washes in paraffin at 60°C, the paraffin was hardened at room temperature and serial sections performed at a thickness of 7 µm (for a detailed protocol, see [41]). Due to the eye pigment present in the sections, the tissue shows a green autofluorescence that was used to visualize the sections. To analyze the neurodegenerative phenotype of different genotypes, we photographed sections that showed the entire optic system and the worst phenotype. For a double blind analysis, pictures were taken without knowing the genotype and numbered. The area of vacuoles in the lamina, medulla, lobula, and lobula plate in one brain hemisphere was then calculated in Photoshop as total pixel number, which was subsequently converted into µm^2^ and then the genotype determined as described in [14]. As controls for *loe*, we originally used flies from the same cross, which did not carry the UAS construct or flies which had a balancer chromosome instead of the additional mutant allele. Because there was no statistically significant difference between the various *loe* controls, we combined all values to one *loe* control for that age. Controls for the AMPKγ knockdown were obtained by crossing the flies to GFP to control for the number of UAS constructs induced by the drivers. *Appl*-GAL4;UAS-LacZ was used as the wild type background in the phototaxis experiments to control for effects caused by the promoter construct and/or by inducing a UAS construct. Statistics was done using GraphPad Prism and one-way ANOVA with a Dunett’s post test when several groups were compared. GraphPad Prism and Student’s Neuman-Keuls test was used when only two groups were compared. The confocal images were obtained from brain whole mounts and the GFP imaged in preparations fixed for 5 min using an Olympus FW1000 confocal microscope.

### Western Blots

Heads from 2–3 d old flies were homogenized as described in [9] and proteins separated on 8% or 12% SDS-PAGE gels and transferred using the Bio-Rad Mini Trans-Blot Cell system. Proteins were transferred to Hybond membranes (Amersham Bioscienses). The antibody against p-cofilin was kindly provided by Buzz Baum (University College London, [42]) and used at 1∶300 and the anti-SSH antibody was kindly provided by the Tadashi Uemura (Kyoto University, [35]) and used at 1∶2000. Anti-cofilin was kindly provided by Michael Goldberg (Cornell University, unpublished). Anti-actin and anti-tubulin were obtained from the Developmental Studies Hybridoma Bank developed under the auspices of the NICHD and maintained by the Department of Biology, University of Iowa, and used at 1∶100. All antibodies were diluted in TBST supplemented with 3% BSA. Bands were visualized using horseradish peroxidase-conjugated secondary antibodies (Jackson ImmunoResearch) at 1∶1000 and the SuperSignal West Pico chemiluminiscent substrate (ThermoScientific). At least three replicates were performed for each experiment. Homogenates from 10 fly heads were used per lane.

### Fast Phototaxis

Fast phototaxis assays were conducted in the dark using the countercurrent apparatus described by [43] and a single light source. A detailed description of the experimental conditions can be found in [44]. Flies were starved overnight, but had access to water and were tested the following morning. Five consecutive tests were performed in each experiment with a time allowance of 6 seconds to make a transition towards the light and into the next vial. A minimum of eight independent tests with groups of 3–6 flies was used per genotype. Statistical analysis was done using GraphPad Prism or one-way ANOVA with Dunett’s post test.

### Visualizing F-actin and G-actin Levels

To visualize the amount of filamentous and globular actin in fly heads, the G-Actin/F-actin In Vivo Assay Biochem Kit from Cytoskeleton was used following the kits manual. An equivalent of 15 heads was loaded per lane. Samples were run on 12% SDS-PAGE gel and Western blots performed as described above. The quantification was done by determining the ratio of F to G for each genotype and then comparing the ratio of *loe* to the ratio of *y w* from the same Western blot (to account for changes in the ratio in the different gels). Similarly, the ratio of F to G from *loe* flies with increased or decreased levels of SSH and TSR were compared to the ratio of loe from the same Western blot. The difference in the ratio is given as % to the control ratio (*y w* or *loe*).

### Cell Culture Experiments

Neuronal cell cultures were prepared from 3rd instar larvae as described in [45]. To determine neurite length photographs were taken after 24 h and 48 h in culture without knowing the genotype, using a Leica inverted microscope. In addition, the number of neurites each cell had extended was counted in the 24 h cultures. The length of the longest neurite of each cell was measured in pixels using ImageJ and converted into µm before the genotype was determined. Measurements of mitochondrial movements were performed on 24 h old primary cultures, using green Mitotracker CM-H2XRos (Molecular Probes, Eugene, USA). Cells were stained for 10 min and then observed with an inverted microscope. Images were taken every 2 seconds for 4.5 minutes. To perform an analysis of mitochondrial movement, we used the tracking function in Metamorph Universal Imaging and created tables with the amount of pixels each mitochondrion moved after 2 seconds. The average distance traveled in the 2 second intervals was determined and converted into velocity. Student’s Neuman-Keuls tests were used to determine significance.

## Supporting Information

Figure S1 *loe* does not show an interaction with MLC. Comparing the area of vacuoles in *loe* also heterozygous for *sqh^AX3^* with *loe* alone did not reveal a difference when analyzed at 5 d or 10 d of age. All flies were females and the SEMs and number of brain hemispheres analyzed, are indicated.
(TIF)

Figure S2 Expressing LIMK does not significantly increase the levels of total cofilin. Head extracts from 5 flies were loaded per lane. A loading control using α-tubulin is shown below.
(TIF)

Figure S3 Expression of LIMK increases F-actin. Limk was induced with *Appl*-GAL4.
(TIF)
