# Supplementary figures and images for: Increased Actin Polymerization and Stabilization Interferes with Neuronal Function and Survival in the AMPKγ Mutant Loechrig
Source: PLoS One. 2014 Feb 25;9(2):e89847. doi: 10.1371/journal.pone.0089847 (PMC3934941; doi:10.1371/journal.pone.0089847)

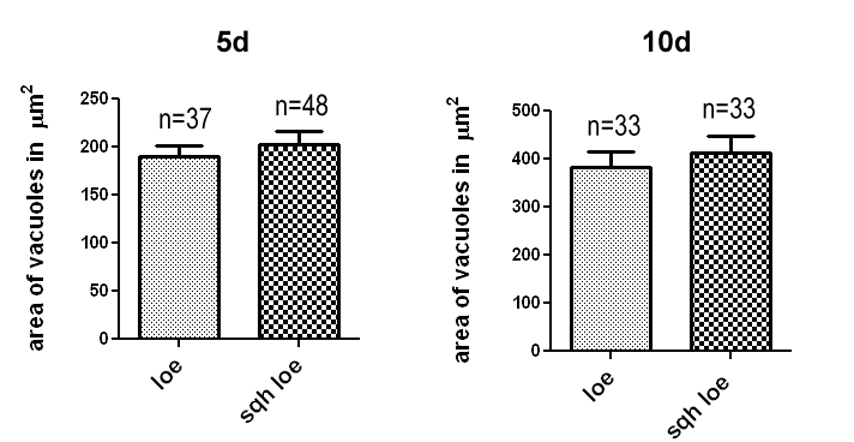

Supplement: Figure S1 — loe does not show an interaction with MLC. Comparing the area of vacuoles in loe also heterozygous for sqhAX3 with loe alone did not reveal a difference when analyzed at 5 d or 10 d of age. All flies were females and the SEMs and number of brain hemispheres analyzed, are indicated. (TIF) [file pone.0089847.s001.tif]

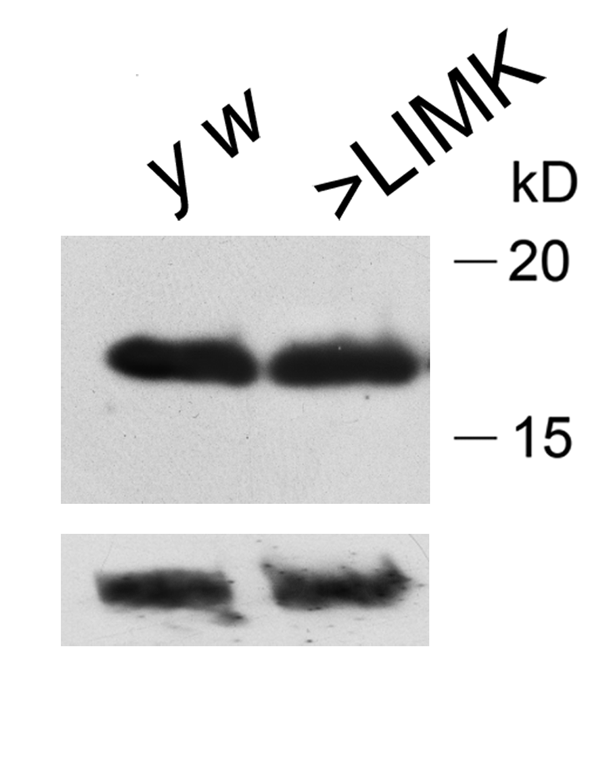

Supplement: Figure S2 — Expressing LIMK does not significantly increase the levels of total cofilin. Head extracts from 5 flies were loaded per lane. A loading control using α-tubulin is shown below. (TIF) [file pone.0089847.s002.tif]

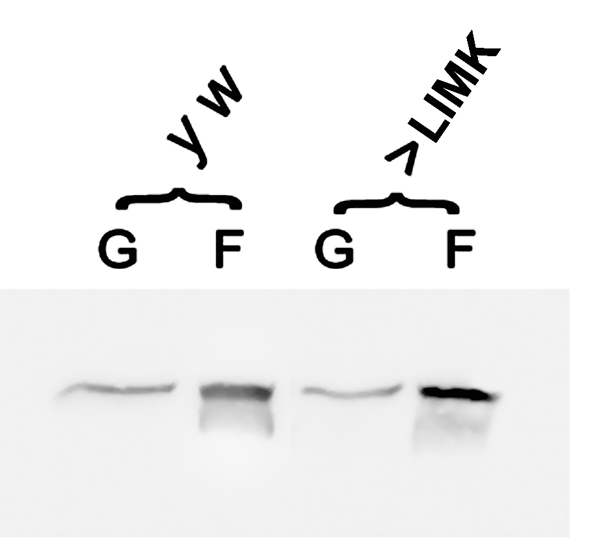

Supplement: Figure S3 — Expression of LIMK increases F-actin. Limk was induced with Appl-GAL4. (TIF) [file pone.0089847.s003.tif]
